# Deficiency of lung-specific claudin-18 leads to aggravated infection with *Cryptococcus deneoformans* through dysregulation of the microenvironment in lungs

**DOI:** 10.1038/s41598-021-00708-6

**Published:** 2021-10-26

**Authors:** Ko Sato, Ikumi Matsumoto, Koya Suzuki, Atsushi Tamura, Aki Shiraishi, Hiroshi Kiyonari, Jun Kasamatsu, Hideki Yamamoto, Tomomitsu Miyasaka, Daiki Tanno, Anna Miyahara, Tong Zong, Takafumi Kagesawa, Akiho Oniyama, Kotone Kawamura, Yuki Kitai, Aya Umeki, Emi Kanno, Hiromasa Tanno, Keiko Ishii, Sachiko Tsukita, Kazuyoshi Kawakami

**Affiliations:** 1 Department of Intelligent Network for Infection Control, Tohoku University Graduate School of Medicine, Sendai, Miyagi Japan; 2 Department of Medical Microbiology, Mycology and Immunology, Tohoku University Graduate School of Medicine, Sendai, Miyagi Japan; 3 Laboratory of Biological Science and Laboratory of Biosciences, Graduate School of Frontier Biosciences, Osaka University, Suita, Osaka Japan; 4 Research Institute for Diseases of Old Age and Department of Clinical Laboratory Medicine, Graduate School of Medicine, Juntendo University, Tokyo, Japan; 5 Laboratory for Animal Resources and Genetic Engineering, RIKEN Center for Biosystems Dynamics Research, Kobe, Japan; 6 Division of Pathophysiology, Department of Pharmaceutical Sciences, Faculty of Pharmaceutical Sciences, Tohoku Medical and Pharmaceutical University, Sendai, Miyagi Japan; 7 Department of Science of Nursing Practice, Tohoku University Graduate School of Medicine, Sendai, Miyagi Japan; 8 Present Address: Center for Transdisciplinary Research, Institute of Research Promotion, Niigata University, Niigata, Japan; 9 Present Address: Department of Clinical Laboratory, Fukushima Medical University, Fukushima, Japan

**Keywords:** Immunology, Microbiology

## Abstract

*Cryptococcus deneoformans* is an opportunistic fungal pathogen that infects the lungs via airborne transmission and frequently causes fatal meningoencephalitis. Claudins (Cldns), a family of proteins with 27 members found in mammals, form the tight junctions within epithelial cell sheets. Cldn-4 and 18 are highly expressed in airway tissues, yet the roles of these claudins in respiratory infections have not been clarified. In the present study, we analyzed the roles of Cldn-4 and lung-specific Cldn-18 (luCldn-18) in host defense against *C. deneoformans* infection. luCldn-18-deficient mice exhibited increased susceptibility to pulmonary infection, while Cldn-4-deficient mice had normal fungal clearance. In luCldn-18-deficient mice, production of cytokines including IFN-γ was significantly decreased compared to wild-type mice, although infiltration of inflammatory cells including CD4^+^ T cells into the alveolar space was significantly increased. In addition, luCldn-18 deficiency led to high K^+^ ion concentrations in bronchoalveolar lavage fluids and also to alveolus acidification. The fungal replication was significantly enhanced both in acidic culture conditions and in the alveolar spaces of luCldn-18-deficient mice, compared with physiological pH conditions and those of wild-type mice, respectively. These results suggest that luCldn-18 may affect the clinical course of cryptococcal infection indirectly through dysregulation of the alveolar space microenvironment.

## Introduction

The two sister species, *Cryptococcus neoformans* (formerly *C. neoformans* var. *grubii*, serotype A) and *C. deneoformans* (formerly *C. neoformans* var. *neoformans*, serotype D), are yeast-type fungal pathogens with thick capsules composed of polysaccharides such as glucuronoxylomannan and galactoxylomannan^1^. These fungi infect the lungs via airborne transmission and cause life-threatening meningoencephalitis in patients with impaired cell-mediated immunity such as acquired immunodeficiency syndrome^2^. As both of these *Cryptococcus* spp. are intracellularly growing yeasts, they are eradicated primarily by the cellular immune mechanism, which is critically regulated by the Th1-Th2 immune balance^2–6^. The Th1-mediated immune response supports host defense by inducing production of nitric oxide (NO), which enhances macrophages’ ability to kill *Cryptococcus*, and by promoting the formation of granulomas at infection sites, which prevents the spread of this fungal pathogen to the surrounding lung tissues and to the CNS^6–9^. The Th2 immune response, in contrast, disturbs host defense by suppressing macrophages’ killing of *Cryptococcus* and granuloma formation^6,7,10^. In a recent study, we demonstrated that IL-17A regulates Th1-mediated host defense against cryptococcal infection^13^; other studies have reported other roles for this cytokine^12–16^.

Claudins (Cldns), cell–cell adhesion molecules with four transmembrane domains located at the tight junctions (TJs) between the cells in epithelial cell sheets, play various roles in the formation of paracellular barriers and channels^17–20^. Twenty-seven members of the Cldn protein family occur in mammals, and each of these subtypes is expressed in an organ-specific and developmental stage-dependent manner^21–23^. Ohta et al. have reported that Cldn-1, 3, 4, 7, and 10 are components of airway TJs, whereas Cldn-5 and 18 are expressed in alveolar TJs^22^. In addition, LaFemina et al. have demonstrated that Cldn-3, 4, and 18 are the predominant claudins expressed in primary rat alveolar epithelial cells^23^. Cldn-4 has been reported to function as a paracellular sodium barrier and to play an undefined role in protecting the lungs from acute injury, although its contribution to normal lung physiology is limited^24^. High expression of Cldn-18 has been described in lung and stomach epithelium, with lung-specific (Cldn-18.1 or luCldn-18) and stomach-specific (Cldn-18.2 or stCldn-18) isoforms of Cldn-18 regulated by alternative promoter usage^25,26^. luCldn18 has been shown to be involved in alveolarization, alveolar epithelial permeability, and alveolar fluid clearance^27,28^. The role of these claudins in respiratory infections has not been clarified, however. To correct this, in the present study, we investigated the role of claudins, especially Cldn-4 and luCldn-18, in host protection and immune response against infection with *C. deneoformans* using mice genetically lacking either Cldn-4 or luCldn-18.

## Materials and methods

### Ethical statement

This study was performed in strict accordance with the Fundamental Guidelines for Proper Conduct of Animal Experiments and Related Activities in Academic Research Institutions under the jurisdiction of the Ministry of Education, Culture, Sports, Science and Technology in Japan, 2006. All experimental procedures involving animals followed the Regulations for Animal Experiments and Related Activities at Tohoku University, Sendai; Osaka University, Osaka; and RIKEN, Japan and were approved by the Institutional Animal Care and Use Committee at Tohoku University, Osaka University, and RIKEN Kobe branch. All experiments were performed under anesthesia, and all efforts were made to minimize the suffering of the animals. The study was carried out in compliance with the ARRIVE guidelines (https://arriveguidelines.org/).

### Establishment of Cldn-4 and lung-specific Cldn-18-deficient mice

The Cldn-4 targeting construct was made using the Pnt1.1 vector, which contains a neomycin-resistance gene (provided by M. Okabe, University of Osaka, Osaka, Japan) (Fig. S1). The 5′ arm and 3′ arm were amplified by PCR using a BacPac vector, which included the Cldn-4 gene from C57BL/6, as the template. The amplified DNA fragments were confirmed by sequencing and inserted into the upstream and downstream regions of the PGK-Neo cassette, respectively. The targeting construct was linearized and electroporated into 129-derived embryonic stem cells. After selection with G418, drug-resistant clones were picked up and screened by PCR and Southern blot analysis. Cldn-4-targeted embryonic stem clone was microinjected into blastocysts derived from C57BL/6 mice and transferred to pseudopregnant females. The resulting chimeric mice were bred with C57BL/6 mice to obtain germline transmitted heterogenous (+/−) mice. The resulting Cldn4^+/−^ mice were intercrossed to obtain Cldn4^−/−^ mice. Genotyping was performed by PCR (CL4-WTF2: 5′-CAGTTGCCCACCTCGTAGCAACGAC-3′, CL4-WTR2; 5′-ATCCACCAGCAATTTGGATGTAAGC-3′, CL4-neo; 5′-CCGGTGGATGTGGAATGTGTGCGAGGCC-3′). The product of the wild-type allele is ~ 300 bp, and the targeted allele yields a ~ 500-bp product. For lung-specific claudin-18 (Cldn18.1)-deficient mice, the chimeric mice were generated as described elsewhere (http://www2.clst.riken.jp/arg/methods.html) and crossed with C57BL/6 mice to obtain heterozygous lung-specific Cldn18.1 flox/wt mice (Accession No. CDB0809K: http://www2.clst.riken.jp/arg/mutant%20mice%20list.html) (Fig. S2). Crossing these mice with CAG-Cre Tg mice^29^ yielded Cldn18.1^+/−^ mice which were then intercrossed to obtain Cldn18.1^−/−^ mice. Genotyping was performed by PCR (Cldn18-F: 5′-GTGAATCCCAGCAAGTTTTGTTATAGA-3′, Cldn18-WTR; 5′-CTCAGTGTTTGCTCATACTGTTTTCCTT-3′, Cldn18-KOR; 5′-GAATGCAAAGTGCCTATGATGC-3′). The product of the wild-type allele is ~ 310 bp, and the targeted allele yields a ~ 640-bp product.

### Mice

Male or female mice at 6 to 8 weeks of age and 16 to 24 g of weight were used in the analyses. Mice were randomly allocated to the various experimental groups. All mice were kept under specific pathogen-free conditions at the Institute for Animal Experimentation, Tohoku University Graduate School of Medicine; the Osaka University Laboratory of Biological Science, Graduate School of Frontier Biosciences and Medicine; or RIKEN Kobe branch. The conditions of the breeding room were as previously described^11,30–35^.

### Inoculation with *Cryptococcus deneoformans*

A strain of *C. deneoformans*, designated as B3501 (a kind gift from Dr. Kwong Chung, National Institute of Health, Bethesda, MD, USA) was used. The yeast cells were cultured on potato dextrose agar (Eiken, Tokyo, Japan) plates for 2–3 d before use. Mice were anaesthetized by an intramuscular injection of 0.3 mg/kg of midazolam (Fuji Phama, Tokyo, Japan) and 0.02 mg/kg of Medetomidine hydrochloride (Nippon Zenyaku Kogyo, Fukushima, Japan) and an intraperitoneal injection of 15 mg/kg of pentobarbital (Abbott Laboratory, North Chicago, IL, USA). Live *C. deneoformans* (1 × 10^6^ cells, 50 μl) was inoculated into the trachea of each mouse using a 24-gauge catheter (Terumo, Tokyo, Japan).

### Preparation of bronchoalveolar lavage fluids and lung interstitial homogenates

Mice were sacrificed at various timepoints after infection. Bronchoalveolar lavage fluids (BALFs) were prepared as previously described^34,35^. After the BALFs were collected, lung homogenates were prepared as previously described^11,30–33^.

### Enumeration of viable *C. deneoformans*

Evaluation of the fungal burdens in the lungs and brains were carried out as previously described^11,30–33^. Briefly, the lungs and brains were homogenized separately in 5 ml (lungs) or 1 ml (brains) of distilled water through a stainless-steel mesh at room temperature. In some experiments, BALFs and lung homogenates after BAL were analyzed separately in 3 ml and 5 ml, respectively, of distilled water. These samples were diluted, inoculated on PDA plates, and cultured before the resulting colonies were counted.

### Histological examination

Histopathological specimens of the lungs were obtained as previously described^11,30–35^. Lung specimens were stained with hematoxylin–eosin (H-E) or periodic acid-Schiff (PAS) at the Biomedical Research Core, Animal Pathology Platform of Tohoku University Graduate School of Medicine. The granulomatous area was calculated for the H&E-stained sections as the proportion of granulomatous area relative to the total lung area using ImageJ software (https://imagej.nih.gov/ij/; provided in the public domain by the National Institutes of Health).

### Measurement of BALF protein

Protein concentration in BALFs was measured using a bicinchoninic acid (BCA) protein assay reagent kit (Thermo Fisher Scientific K.K., Yokohama, Japan). The detection limit was 125 μg/mL.

### Flow cytometry

The BALF cells were washed three times in PBS containing 1% fetal calf serum (FCS) and 0.1% sodium azide and then stained with APC-anti-CD3ε mAb (clone 145-2C11; BioLegend), FITC-anti-CD4 mAb (clone GK1.5; BioLegend), APC/Cy7-anti-CD8 mAb (clone 53–6.7; BioLegend), and PE/Cy7-anti-NK1.1 mAb (clone PK136; BioLegend), or FITC-anti-CD11c mAb (clone N418; BioLegend), PE-anti-CD11b mAb (clone M1/70; BioLegend), PE/Cy7-anti-Gr-1 mAb (clone RB6-8C5; BioLegend), and Pacific Blue-anti-F4/80 mAb (clone BM8; BioLegend). Isotype-matched IgG was used for control staining. The stained cells were analyzed using a BD FACS Canto II flow cytometer (BD Bioscience) as previously described^11,31–33^.

### Cytokine assay

BALFs were collected in 1 ml PBS. After BAL, the lungs were excised and then homogenized separately in 5 ml PBS through a stainless-steel mesh. After centrifugation, the supernatants were collected and stored at − 70 °C before use. Concentrations of each cytokine in BALF and lung homogenates were measured using an ELISA kit as previously described^11,30–35^.

### Measurement of ion concentration and pH in BALFs

BALFs were collected in 1 ml saline from 8-week-old mice under deep anesthesia. Na^+^ and K^+^ ion concentrations in BALFs were directly measured using Na^+^ and K^+^ compact ion meters (Horiba, Kyoto, Japan) according to the manufacturer’s instructions. Cl^−^ ion concentrations in BALFs diluted with MQ water were measured using the QuantiChrom Chloride Assay Kit (BioAssay Systems, Hayward, CA, USA). The electrical potentials were measured using the LAQAtwin compact pH meter (Horiba, Kyoto, Japan) according to the manufacturer’s instructions. A standard curve was prepared from the electrical potential of saline adjusted to pH 4, 7, and 9 with HCl or NaOH. The pH values of the BALF samples were calculated from this standard curve after the measured values were corrected for the electrical potential of saline.

### In-vitro culture of *C. deneoformans*

*C. deneoformans* was cultured on PDA plates for two to three days before use. The yeast cells were suspended in RPMI 1640 medium with 20 mM HEPES and without NaHCO_3_ (Sigma-Aldrich, St. Louis, MO, USA), prepared at 1 × 10^4^ cells/ml, and cultured at 37 °C for 24 h at different pH values (6.8 and 7.3). The number of organisms was counted using a hemocytometer. The yeast cells were concentrated onto microscope slides using a cytospin, stained with PAS (Muto Pure Chemicals, Tokyo, Japan), and observed under a microscope. The budding ratio of each PAS-stained specimen was estimated as the number of budding yeast cells out of 200 cells.

### Cell division assay

*C. deneoformans* was cultured on PDA plates for two to three days before use. The yeast cells were stained with 50 μM carboxyfluorescein succinimidyl ester (CFSE) for 30 min in 10 ml PBS at 30 °C, and washed three times in PBS to remove excess CFSE. CFSE-labeled *C. deneoformans* was used for in vitro culture and in vivo infection by the method described above. The mean fluorescent intensity (MFI) of CFSE in the cultured yeast cells or CD45^−^ cells in BALFs after infection was analyzed using a BD FACS Canto II flow cytometer.

### Statistical analysis

Data were analyzed using JMP Pro 11.2.0 software (SAS Institute Japan, Tokyo, Japan). Data are expressed as mean ± SD. Differences between groups were examined for statistical significance using Welch’s *t*-test. A *p* value of less than 0.05 was considered significant.

## Results

### Effect of claudin deficiencies on host protection and inflammatory response against cryptococcal infection

**Figure 1:**
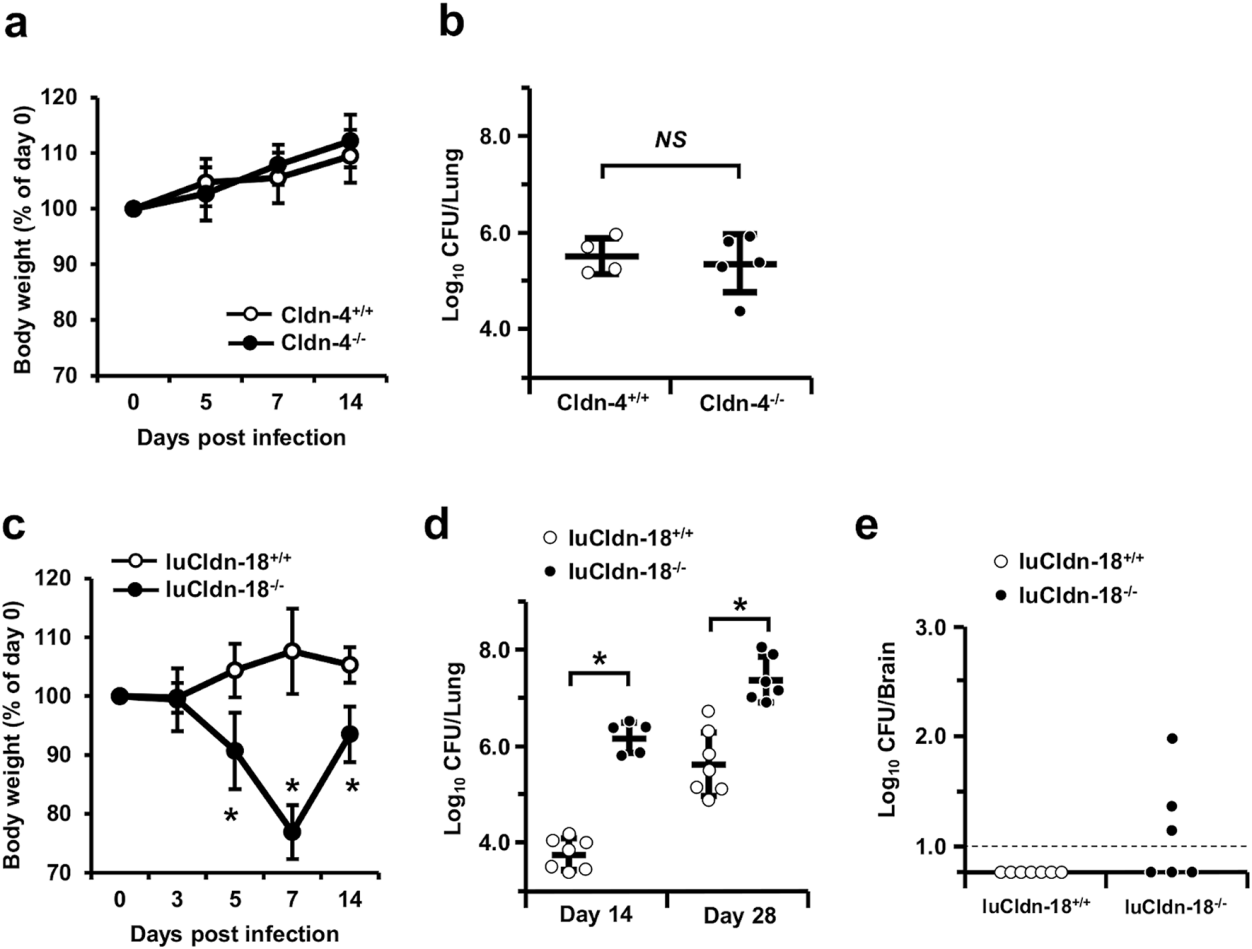
Effect of claudin deficiencies on *C. deneoformans* infection. (**a**, **b**) Cldn-4^+/+^ (n = 4) and Cldn-4^−/−^ mice (n = 5) were infected intratracheally with *C. deneoformans.* (**a**) Mouse weight is presented as weight relative to the initial weight of each mouse. (**b**) The numbers of live colonies in the lungs were counted on day 14 post-infection. Each symbol represents a separate mouse, and bars indicate the mean ± standard deviation (SD). (c-e) luCldn-18^+/+^ (day 14, n = 7; day 28, n = 7) and luCldn-18^−/−^ mice (day 14, n = 5; day 28, n = 6) were infected intratracheally with *C. deneoformans.* (**c**) Mouse weight is presented as weight relative to the initial weight of each mouse. The numbers of live colonies in the lungs on days 14 and 28 (**d**) and in the brains on day 28 post-infection (**e**) were counted. Each symbol represents a separate mouse, and bars indicate the mean ± SD. The dashed line indicates the detection limit. *, *p* < 0.05. *NS*, not significant. There were no mice excluded from this analysis.

**Figure 2:**
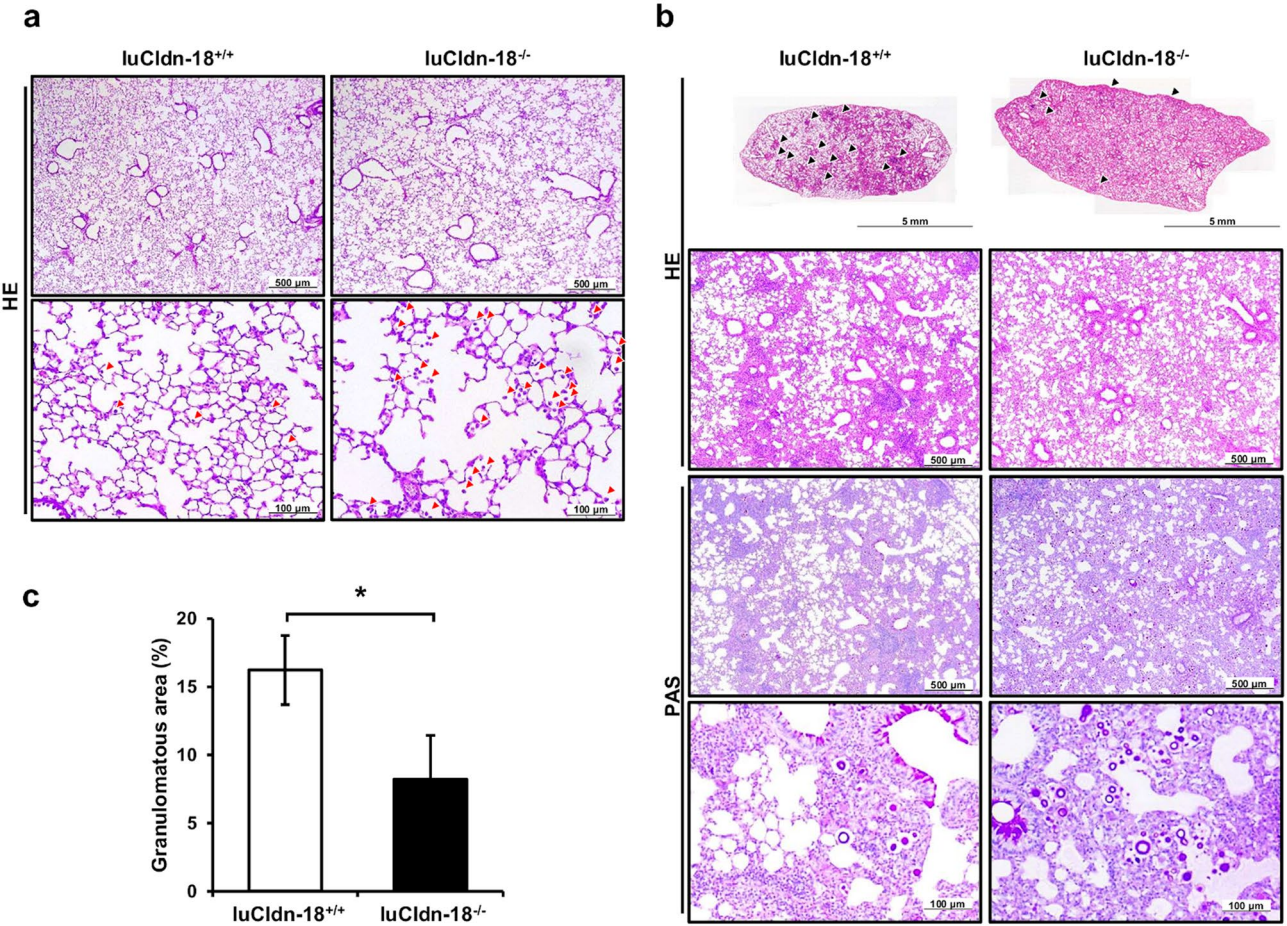
Effects of luCldn-18 deficiency on pulmonary histological findings after infection with *C. deneoformans.* (**a**) Lung sections of uninfected luCldn-18^+/+^ (n = 3) and luCldn-18^−/−^ mice (n = 3) were stained with H-E and observed under a light microscope at ×40 (upper panels) and ×200 (under panels)*.* Red arrows show alveolar macrophages. (**b**) luCldn-18^+/+^ and luCldn-18^−/−^ mice were infected intratracheally with *C. deneoformans.* Sections of the lungs on day 14 post-infection were stained with H-E or PAS and observed under a light microscope. Original magnifications: upper panels, the upper lobe of the left lungs; middle panels, ×40; lower panels, ×200. Representative pictures are shown. Black arrows show granulomas. (**c**) The proportions of granulomatous areas were calculated. Each column represents the mean ± SD of 3 mice. *, *p* < 0.05.

To explore the effects of Cldn-4 and luCldn-18 deficiency on host defense against cryptococcal infection, Cldn-4-deficient mice, luCldn-18-deficient mice, and control mice for each claudin-deficient genotype were infected with *C. deneoformans*, and the growth of this fungal pathogen in the lungs and brains was examined. After cryptococcal infection, mouse body weights were not lower in Cldn-4^−/−^ mice compared with Cldn-4^+/+^ mice (Fig. 1a). In addition, the fungal burdens in the lungs were not significantly different between Cldn-4^+/+^ and Cldn-4^−/−^ mice on day 14 post-infection (Fig. 1b). In contrast, the lung and brain burdens of yeast cells were higher in luCldn-18^−/−^ mice than in luCldn-18^+/+^ mice on days 14 and 28 post-infection (Fig. 1c-e). We also conducted a histological analysis to determine how luCldn-18 deficiency affected the inflammatory response in the lungs after cryptococcal infection. Defective alveolar formation and increased alveolar macrophage counts were observed in uninfected luCldn-18^−/−^ mice compared to luCldn-18^+/+^ mice (Fig. 2a). In addition, massive multiplication of yeast cells with poor granulomatous responses was observed in luCldn-18^−/−^ mice, whereas luCldn-18^+/+^ mice showed markedly fewer yeast cells with granulomatous responses (Fig. 2b, c).

**Figure 3:**
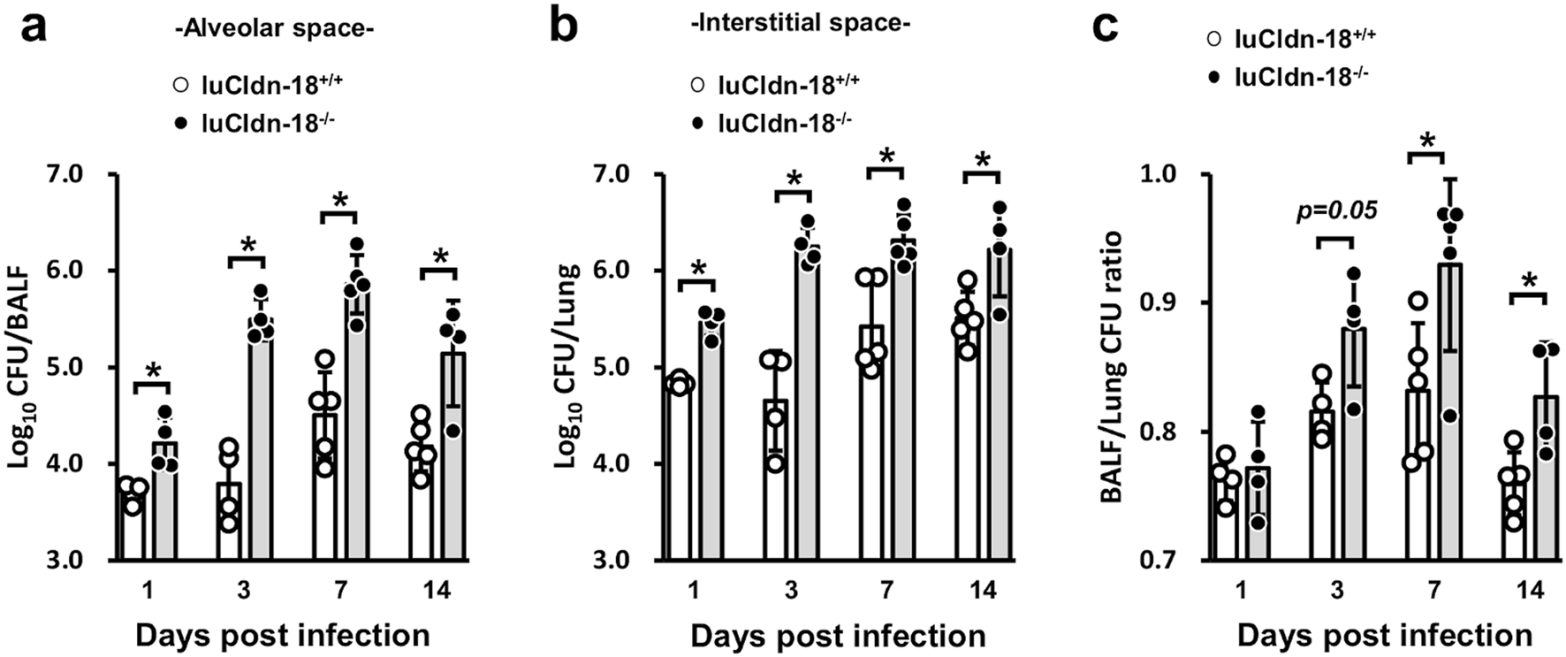
Effect of luCldn-18 deficiency on the localization of *C. deneoformans.* luCldn-18^+/+^ (day 1, n = 4; day 3, n = 4; day 7, n = 5; day 14, n = 5) and luCldn-18^−/−^ mice (day 1, n = 4; day 3, n = 4; day 7, n = 5; day 14, n = 4) were infected intratracheally with *C. deneoformans.* The numbers of live colonies in BALFs (**a**, alveolar space) and lung interstitial homogenates after BAL (**b**, interstitial space) on days 1, 3, 7, and 14 post-infection were counted. (**c**) The ratio of the number of live colonies in BALFs to the number in the lungs was calculated for each mouse. Each symbol represents a separate mouse, and bars indicate the mean ± SD. *, *p* < 0.05. There were no mice excluded from this analysis.

To examine the localization of the fungus in the lungs, we compared the numbers of live *C. deneoforman*s colonies in BALFs (alveolar space) and lung homogenate after BAL (interstitial space) between luCldn-18^+/+^ and luCldn-18^−/−^ mice. The fungal burdens in both BALFs and lung tissue after BAL were greater in luCldn-18^−/−^ mice than in luCldn-18^+/+^ mice on days 1, 3, 7, and 14 post-infection (Fig. 3a, b). In particular, the increase in the fungal burden in the alveolar space of luCldn-18^−/−^ mice was remarkable on days 3, 7, and 14 post-infection (Fig. 3c).

### Effect of luCldn-18 deficiency on the immune response after infection with *C. deneoformans*

**Figure 4:**
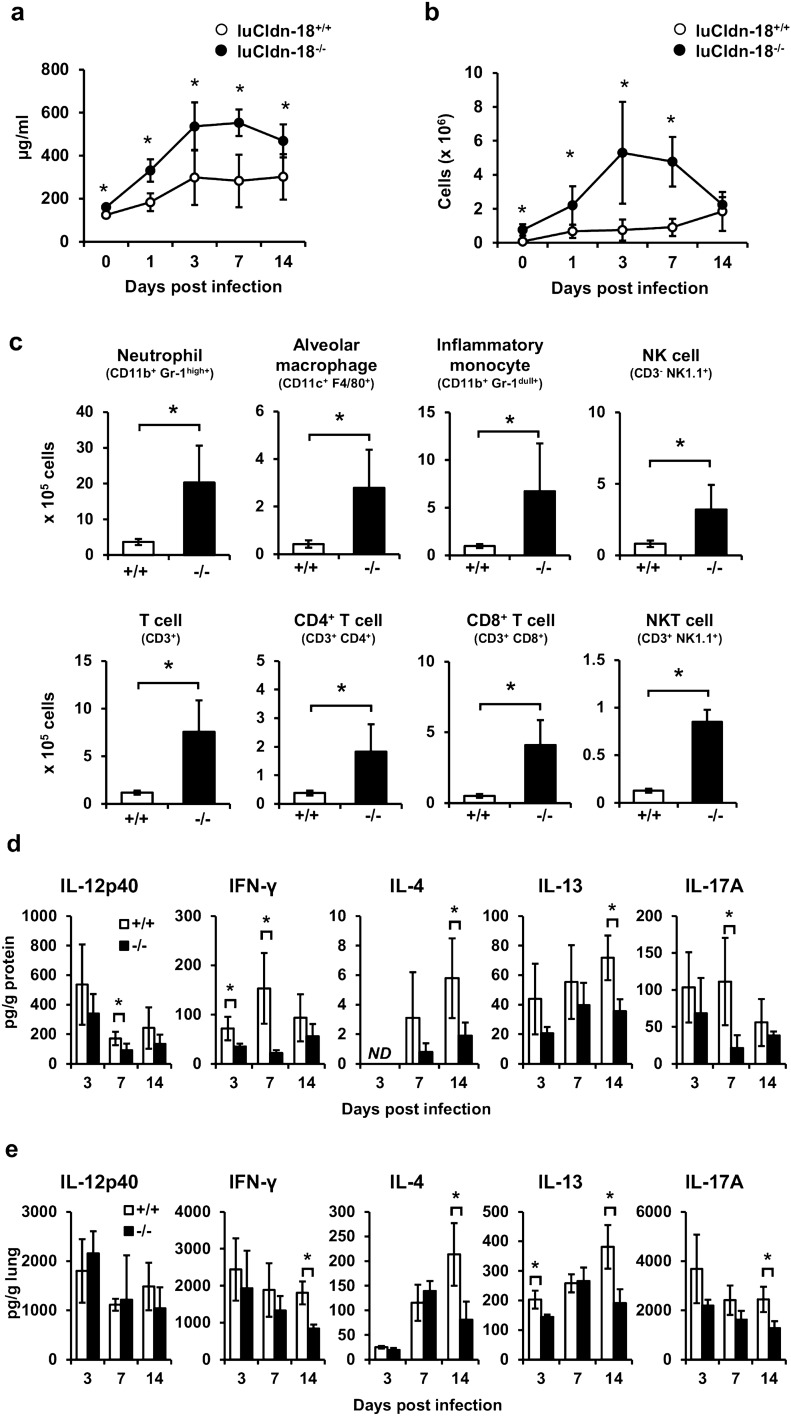
Effects of luCldn-18 deficiency on inflammatory response in BALFs after infection with *C. deneoformans.* luCldn-18^+/+^ (day 0, n = 4; day 1, n = 5; day 3, n = 5; day 7, n = 6; day 14, n = 6) and luCldn-18^−/−^ mice (day 0, n = 4 ;day 1, n = 4; day 3, n = 6; day 7, n = 6; day 14, n = 7) were infected intratracheally with *C. deneoformans.* Protein concentration (**a**) and cell counts (**b**) in BALFs were measured on days 0 (uninfected), 1, 3, 7, and 14 post-infection. Each column represents the mean ± SD. (**c**) BALF cells were prepared on day 3 post-infection. The cell population was analyzed using flow cytometry, and the number in each subset was calculated. Each column represents the mean ± SD. Cytokine production in BALF (**d**) and lung interstitial homogenates after BAL (**e**) at the indicated time intervals after *C. deneoformans* infection. Each column represents the mean ± SD. +/+, luCldn-18^+/+^ mice. −/−, luCldn-18^−/−^ mice. *ND*, not detected. *, *p* < 0.05. There were no mice excluded from this analysis.

During infection with various microorganisms, an inflammatory response is induced, increasing total protein concentrations and inflammatory cell counts^36^. The next series of experiments examined the effect of luCldn-18 deficiency on the immune response to *C. deneoformans*. Total protein concentrations and cell counts in BALFs were significantly greater in luCldn-18^−/−^ mice than in luCldn-18^+/+^ mice on days 0, 1, 3, 7, and 14 post-infection (Fig. 4a, b).

The distributions of various cell types in the BALF were analyzed using flow cytometry on day 3 post-infection (when cell counts peaked). The numbers of neutrophils, alveolar macrophages, inflammatory monocytes, NK, CD4^+^T, CD8^+^T, and NKT cells were significantly greater in luCldn-18^−/−^ mice than in luCldn-18^+/+^ mice (Fig. 4c). Host defense against cryptococcal infection is largely regulated by the balance between the Th1 and Th2 immune responses^2–7^. Our previous study showed that IL-17A is involved in the negative regulation of local host defenses against *C. deneoformans* infection through suppression of the Th1 response^11^. Here, therefore, we examined the effect of luCldn-18 deficiency on cytokine production in response to *C. deneoformans* infection by measuring cytokine concentrations in BALFs and lung homogenate after BAL on days 3, 7, and 14 post-infection. Production of IFN-γ in BALF on days 3 and 7 and in lung homogenate after BAL on day 14 were significantly lower in luCldn-18^−/−^ mice than in luCldn-18^+/+^ mice (Fig. 4d, e). Similarly, production of IL-4 and IL-13 in BALF on day 14 and in the lungs on days 3 and 14 were significantly lower in luCldn-18^−/−^ mice than in luCldn-18^+/+^ mice (Fig. 4d, e). In addition, production of IL-17A in BALF on day 7 and in the lungs on day 14 were significantly lower in luCldn-18^−/−^ mice than in luCldn-18^+/+^ mice (Fig. 4d, e).

### Effect of luCldn-18 deficiency on homeostasis of the lung microenvironment

**Figure 5:**
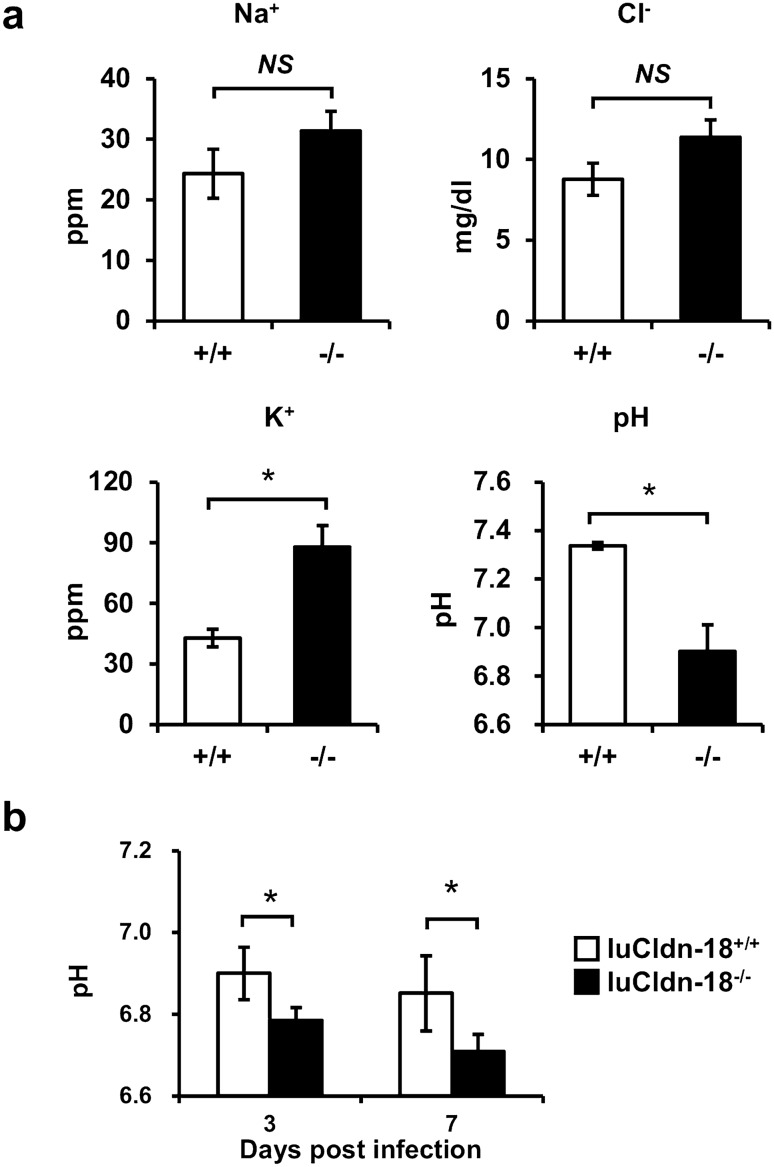
Effects of luCldn-18 deficiency on ion concentration and pH in BALFs. (**a**) The concentrations of several ions (Na^+^, Cl^−^, K^+^) and pH were measured in BALFs of uninfected luCldn-18^+/+^ (n = 3) and luCldn-18^−/−^ mice (n = 3)*.* Each column represents the mean ± SD. (**b**) luCldn-18^+/+^ (n = 4) and luCldn-18^−/−^ mice (n = 4) were infected intratracheally with *C. deneoformans.* pH in BALFs was measured on day 3 and 7 post-infection. Each column represents the mean ± SD. *, *p* < 0.05. *NS*, not significant. There were no mice excluded from this analysis.

luCldn-18 is involved in alveolar fluid homeostasis by regulating solute and ion permeability between alveolar epithelial cells^27^. The next series of experiments examined the effect of luCldn-18 deficiency on the homeostasis of the lung microenvironment. In a steady state, K^+^ ion concentration in BALFs was significantly higher in luCldn-18^−/−^ mice than in luCldn-18^+/+^ mice, while pH was significantly lower (Fig. 5a). Airway surface liquid pH can be altered by disease and lung inflammation, in part because airway inflammation promotes acidification^37^. Alveolus acidification was observed after cryptococcal infection in both genotypes (Fig. 5b). In addition, alveolar pH remained significantly lower in luCldn-18^−/−^ mice than in luCldn-18^+/+^ mice until at least seven days after infection (Fig. 5b).

### Effect of luCldn-18 deficiency on fungal growth

**Figure 6:**
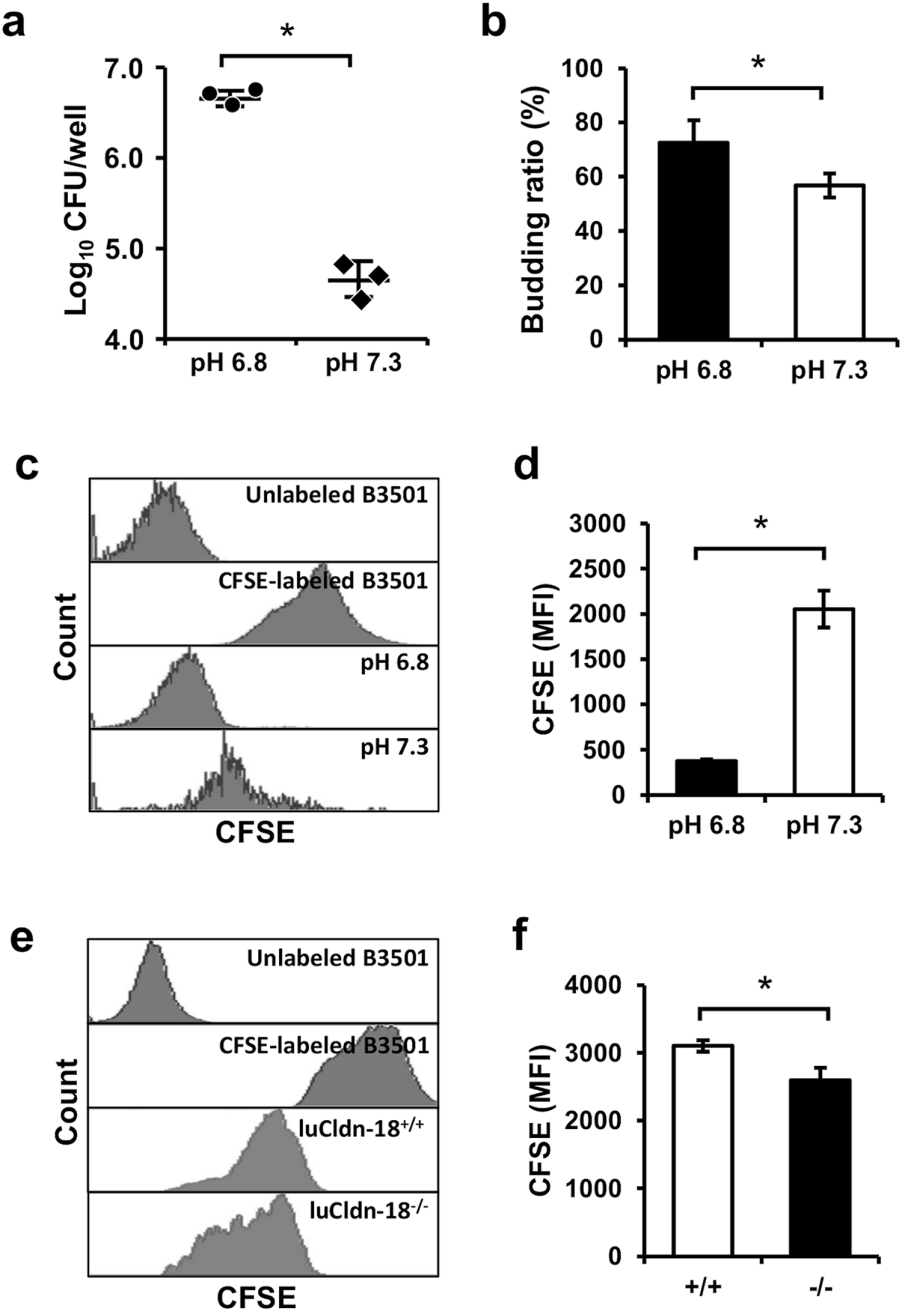
Effect of luCldn-18 deficiency on fungal growth. *C. deneoformans* was cultured in RPMI 1640 medium containing 20 mM HEPES at 37 °C for 24 h at different pH values (6.8 and 7.3, n = 3 cultures for each condition). (**a**) The number of organisms was counted using a hemocytometer. Each symbol represents a separate culture, and bars indicate the mean ± SD. (**b**) The budding ratio was calculated in the PAS-stained specimens. Each column represents the mean ± SD. CFSE-labeled *C. deneoformans* was cultured in RPMI 1640 medium containing 20 mM HEPES at 37 °C for 24 h at different pH values (6.8 and 7.3) and cell division was analyzed using flow cytometry (**c**, **d**). Representative histograms (**c**) and the MFI of CFSE (**d**) are shown. CFSE-labeled *C. deneoformans* cultured in PDA plates was used as a positive control. Each column represents the mean ± SD. luCldn-18^+/+^ (n = 3) and luCldn-18^−/−^ mice (n = 3) were infected intratracheally with CFSE-labeled *C. deneoformans* (**e**, **f**)*.* BALF cells were prepared at 24 h post-infection. CD45^−^CFSE^+^ cells in the alveolar space were defined as fungal cells and analyzed for CFSE fluorescence intensity using flow cytometry. (**e**) Representative histograms are shown. CFSE-labeled *C. deneoformans* cultured in PDA plates was used as a positive control. (**f**) The MFI of CFSE was calculated. Each column represents the mean ± SD. *, *p* < 0.05. There were no mice excluded from this analysis.

Cryptococcal replication has been reported to be enhanced at acidic pH as opposed to physiological pH^38,39^. In this study, similarly, the numbers of yeast cells and the budding rate after in vitro culture were significantly greater at BALF’s pH condition of luCldn-18^−/−^ mice (pH 6.8) than in those of luCldn-18^+/+^ mice (pH 7.3) (Fig. 6a, b). In addition, in the cell division assay using CFSE, CFSE labeling of yeast cells after in vitro culture, which is correlated with cell propagation, was significantly lower at pH 6.8 than at pH 7.3 (Fig. 6c, d). In our in vivo infection experiment, likewise, CFSE expression on CD45^−^ cells in BALFs after cryptococcal infection was significantly lower in luCldn-18^−/−^ mice than in luCldn-18^+/+^ mice (Fig. 6e, f), suggesting that cryptococcal replication was increased at the acidic pH that occurs in luCldn-18^−/−^ mice as opposed to the physiological pH that occurs in luCldn-18^+/+^ mice.

## Discussion

TJs function as the primary structural component controlling paracellular permeability and as barriers in the spaces between the alveolar epithelial cells^40^, yet their contribution to respiratory infection has not been thoroughly analyzed. Cldn-4 and luCldn-18 are among the components of airway TJs^22,23^. In the present study, luCldn-18 deficiency increased susceptibility to pulmonary *C. deneoformans* infection, while Cldn-4 deficiency did not affect fungal clearance. TJs assemble through homotypic and heterotypic cis- and trans-interactions between claudins in paracellular localization^41,42^. In lung epithelial cells, Kage et al. found that Cldn-4 deficiency did not alter the expression of other Cldns (Cldn-3, 5, 7, and 18) or ion permselectivity at steady state^24^. In alveolar epithelial cells, in contrast, Li et al. demonstrated that luCldn-18 deficiency increased expression of Cldn-3 and Cldn-4 in whole lungs and ion permeability at steady state^27^. In addition, impaired alveolarization was observed in luCldn-18-deficient mice^28^. In the current study, likewise, luCldn-18 deficiency led to altered expression of other Cldns (Cldn-3 and 5) in the lungs, with increased K^+^ ion concentration, decreased pH in BALF, and impaired alveolar formation. These data suggest that luCldn-18 may have an important role in maintaining homeostasis of the microenvironment within alveolar spaces and that its impairment may increase susceptibility to cryptococcal infection.

luCldn18 deficiency increased fungal burden not only in the lungs but also in the brain. The blood–brain barrier (BBB) protects the central nervous system (CNS) by restricting the passage of molecules and microorganisms^43^. It has been suggested that *C. deneoformans* may enter the blood or brain from the lung through a Trojan horse mechanism, crossing the BBB as a passenger inside host phagocytes^44^. The increased fungal dissemination into the brain in luCldn-18-deficient mice may be related to pressure changes due to the increased fungal burden in the lungs. Berndt et al. have reported that Cldn-5, 11, 12, and 25 are expressed in murine brain capillaries^45^, and some pathogens are known to infiltrate the CNS by regulating the expression of these Cldns^46^: human immunodeficiency virus type 1 (HIV-1), for example, has been shown to disrupt BBB integrity via modification of Cldn-5, thereby allowing HIV-1 to enter the brain^47^. Group B streptococcus disrupts the paracellular pathway of the BBB by downregulating claudin-5 via upregulation of Snail1 and enhances bacterial invasion into the CNS^48^. Yet the effect on fungal passage of the expression of these Cldns within the brain has not been analyzed. Since the present study did not analyze whether the expression of Cldns in the brain was altered in luCldn-18-deficient mice, further investigations are required to address the possibility that the passage of fungus, including phagocytosed fungus, might be enhanced in luCldn-18-deficient mice due to altered Cldn expression patterns in their brains.

In luCldn-18-deficient mice, the increased population of *C. deneoformans* was more remarkable in the alveolar space than in the lung interstitium. In addition, acidification in the alveolar spaces of luCldn-18-deficient mice was observed during infection with this fungal pathogen. In previous studies, stCldn-18-deficient mice have been found to have lower pH in the stomach due to gastric acid leakage, leading to gastritis and gastric tumorigenesis^18,49,50^. Alveolar macrophages, which constitute a sizable fraction of alveolar subphase fluids, are a substantial source of metabolic H^+^ production under physiological conditions^37^. In the current study, the number of alveolar macrophages was significantly greater in luCldn-18^−/−^ mice than in luCldn-18^+/+^ mice after cryptococcal infection, suggesting that acidification in the alveolar spaces of luCldn-18-deficient mice may be caused by increased production of metabolic H^+^ by alveolar macrophages. In addition, the enhanced cell destruction of increased numbers of macrophages and airway epithelial cells may have induced high K^+^ ion concentrations in alveolar subphase fluids, although our limited analysis of this matter prevents us from drawing firm conclusions.

A Th1-mediated immune response contributes toward the eradication of *C. deneoformans* through inducing macrophage activation and accelerating the formation of granuloma at the infection sites^6–9^, whereas the Th2-mediated immune response counteracts these host protective responses, worsening infection^6,7,10^. In the present study, the production of Th1-related cytokines such as IL-12p40 and IFN-γ was significantly decreased in luCldn-18^−/−^ mice compared to luCldn-18^+/+^ mice after cryptococcal infection. Unexpectedly, however, Th2 cytokines such as IL-4 and IL-13, which suppress host defense against cryptococcal infection by inhibiting macrophage activation and enhancing mucin production^6,7,10,30^, were not increased but rather also decreased in luCldn-18^−/−^ mice compared to luCldn-18^+/+^ mice. In addition, the synthesis of IL-17A, which is involved in the negative regulation of local host defense against *C. deneoformans* infection^11^, was significantly lower in luCldn-18^−/−^ mice than in luCldn-18^+/+^ mice. Thus, the changes in production of host defense-related cytokines were not consistent with the impaired fungal elimination observed in luCldn-18^−/−^ mice. Similar inconsistent results were observed in the infiltration of inflammatory cells including CD4^+^ T cells into the alveolar spaces, which was drastically increased in luCldn-18^−/−^ mice compared to luCldn-18^+/+^ mice. There are no reports of a single luCldn-18 deficiency leading to increasing paracellular cell transport. In the current study, luCldn-18 deficiency induced Cldn expression changes, but the expression of many TJ proteins, including occludin, was maintained. Therefore, the possibility of increased mechanical paracellular transport through alveolar epithelial cells is thought to be low. Previously, Leblebicioglu and co-workers reported that chemotactic responses of human peripheral blood polynuclear leukocytes were significantly increased under decreased pH conditions in in vitro cultures^51^. Lower pH induces activation of monocytes/macrophages and dendritic cells but suppresses T cell immune responses^36,52,53^. Higher levels of extracellular K^+^ ions are reported to suppress the activation of T cell-mediated immune responses^54,55^, which is also observed under the luCldn-18-deficient condition in the current study. These findings suggest that acidification in the alveolar spaces of luCldn-18^−/−^ mice may promote the infiltration of inflammatory cells into the alveolar space while also suppressing the T cell immune response to *C. deneoformans* through acidification and increased levels of K^+^ ions.

In the current study, fungal replication of *C. deneoformans* was significantly enhanced both in acidic culture conditions in vitro and in the alveolar spaces of luCldn-18-deficient mice in vivo compared with physiological pH and control mice, respectively. Several investigators have demonstrated that acidic conditions enhance cryptococcal growth while alkaline conditions inhibit it^37,38,56,57^. Islam et al. have reported that the replication of *Cryptococcus* including the B3501 strain was increased at acidic pH compared to physiological pH conditions^56^. Levitz et al. have reported that *C. neoformans* is able to grow at pH values ranging from 5 to 8, with optimal growth at pH 5^57^. Meanwhile, Vidotto et al. have demonstrated that K^+^ ion concentration does not affect *C. neoformans* phenoloxidase activity, which has long been known as a virulence factor^58^. Collectively, these results suggest that acidification in the alveolar spaces of luCldn-18^−/−^ mice may not only suppress T cell activation but also enhance the growth of the fungal pathogen *C. deneoformans*.

Diseases in which the expression of luCldn-18 changes are not well known, such as diabetes mellitus, hematological diseases and collagen diseases, are known to be risk factors for cryptococcosis in non-HIV patients^59^. In addition, luCldn-18 may be impaired by inflammatory responses and tissue damage induced after cryptococcal infection, but the present study did not analyze whether the expression of luCldn-18 in the lungs was decreased after infection. Analysis of luCldn-18 gene mutations and expression in high-risk patients and luCldn-18 injury mechanisms involved in cryptococcal infection may be useful in finding therapies for infection with this pathogen.

**Figure 7:**
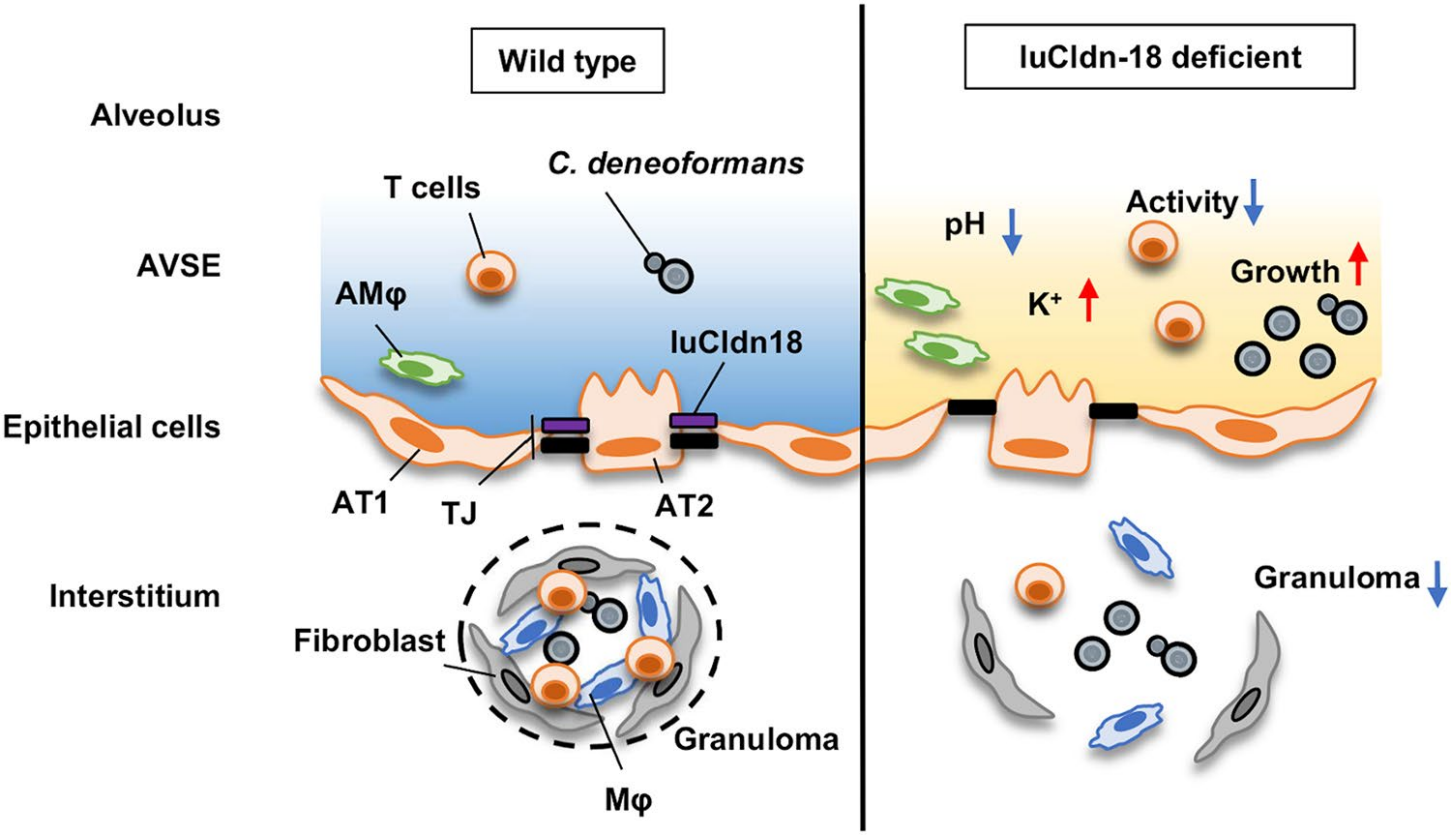
Summary of the role of luCldn-18 in host defense against infection with *C. deneoformans*. luCldn-18 deficiency induces acidification and high K^+^ ion concentration in the alveolar space. Alveolar acidification and high K^+^ ion concentrations inhibit the activation of T cells in response to cryptococcal infection and thereby suppress granuloma formation. In addition, the increased replication efficiency of this fungus at low pH values increases the fungal burden in luCldn-18-deficient mice. Consequently, luCldn-18 indirectly helps prevent cryptococcal infection by maintaining homeostasis of the alveolar space microenvironment. AVSE, alveolar subphase fluid. TJ, tight junction. AMφ, alveolar macrophages. AT1, alveolar type I cells. AT2, alveolar type II cells. This image was drawn by Ko Sato.

In conclusion, the present study demonstrates that luCldn-18 deficiency probably suppresses T cell activation during cryptococcal infection through acidification and increased extracellular K^+^ ion concentration in the alveolar spaces. As *C. deneoformans* has greater replication efficiency in lower pH conditions, the acidification of the alveolar spaces may increase the lung burden of this fungal pathogen in luCldn-18^−/−^ mice. Thus, luCldn-18 may be indirectly involved in limiting the progression of cryptococcal infection in the lungs through maintaining the homeostatic condition in the alveolar space microenvironment, as shown in Fig. 7. The current findings offer a novel approach to and a possibly better understanding of the pathogenic mechanism of cryptococcal infection at the mucosal surfaces of the bronchoalveolar spaces.

## Supplementary Information

Supplementary Information.

## Abbreviations

Cldn: Claudin
luCldn-18: Lung-specific Cldn-18
stCldn-18: Stomach-specific Cldn-18
TJ: Tight junction
PDA: Potato dextrose agar
PBS: Phosphate buffered saline
BALF: Bronchoalveolar lavage fluid
H-E: Hematoxylin–eosin
PAS: Periodic acid-Schiff
BCA: Bicinchoninic acid
FCS: Fetal calf serum
CFSE: Carboxyfluorescein succinimidyl ester
BBB: Blood–brain barrier
CNS: Central nervous system
HIV-1: Human immunodeficiency virus type 1
MFI: Mean fluorescent intensity
AVSF: Alveolar subphase fluid
SD: Standard deviation
ND: Not detected
NS: Not significant
AMφ: Alveolar macrophages
AT1: Alveolar type I cells
AT2: Alveolar type II cells
