# Supplementary material for: Deficiency of lung-specific claudin-18 leads to aggravated infection with Cryptococcus deneoformans through dysregulation of the microenvironment in lungs
Source: Sci Rep. 2021 Oct 26;11:21110. doi: 10.1038/s41598-021-00708-6 (PMC8548597; doi:10.1038/s41598-021-00708-6)
Supplement: Supplementary file 1 — Supplementary Information. [file 41598_2021_708_MOESM1_ESM.pdf]

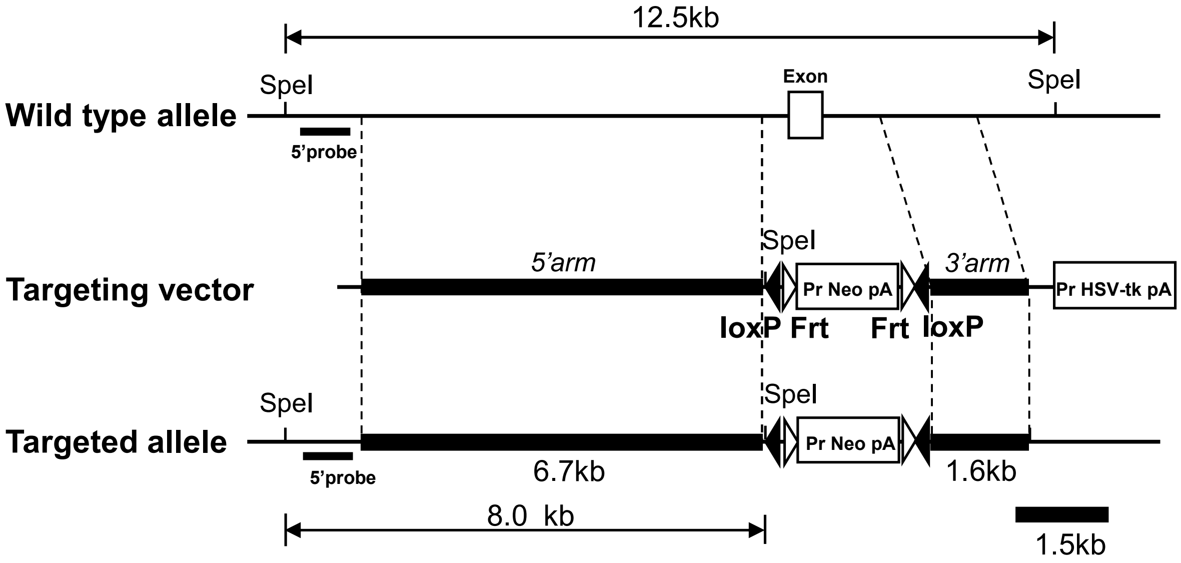

Fig. S1 Generation of Cldn-4-deficient mice. Construction of the wild-type allele, targeting vector, and targeted allele of the mouse Cldn-4 gene.

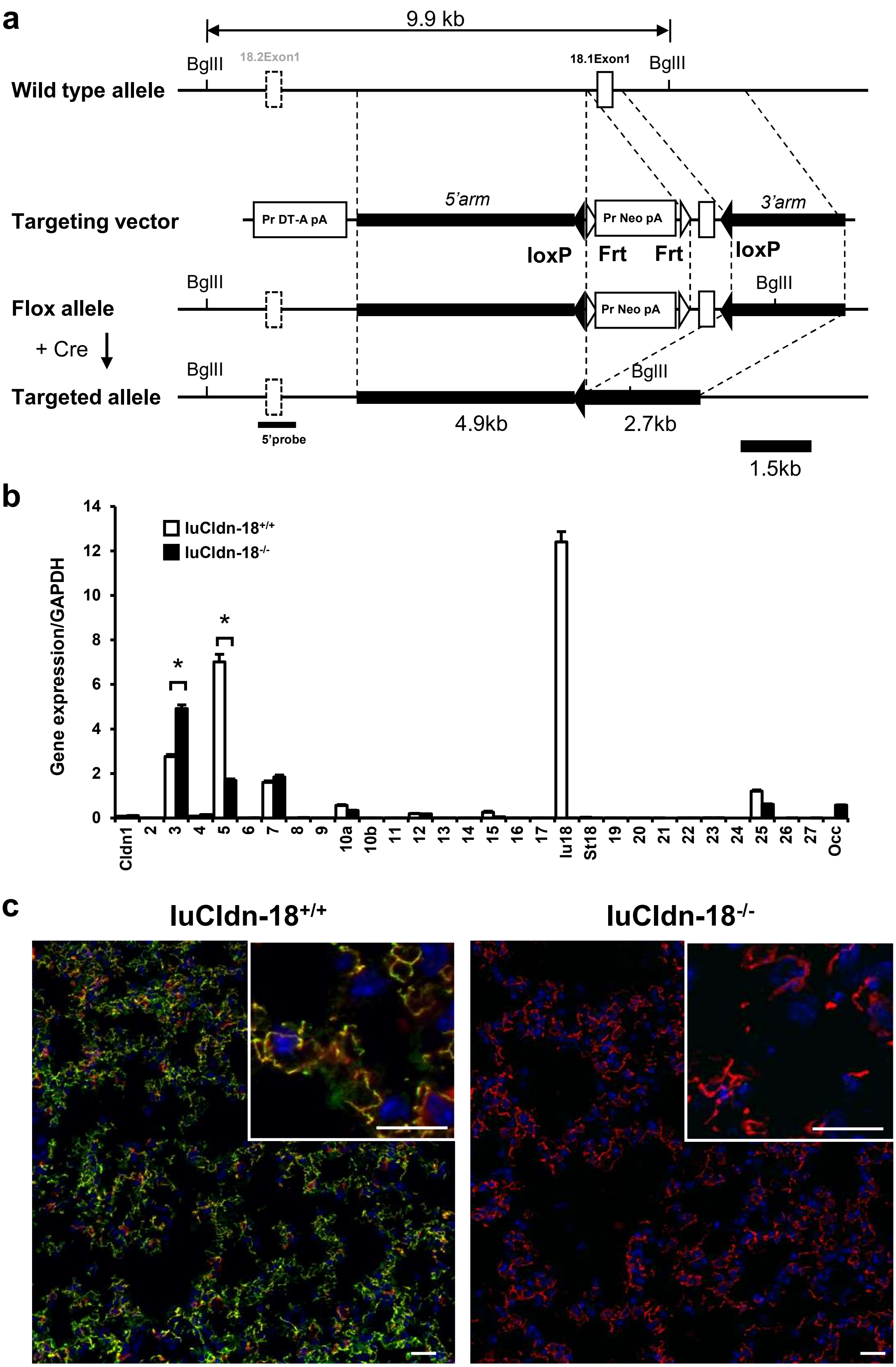

Fig. S2 Generation of luCldn-18-deficient mice. (A) Construction of the wild-type allele, targeting vector, Flox allele, and targeted allele of the mouse Cldn-18 gene. (B) Expression levels of Cldns and Occludin (Occ) in the lungs from luCldn-18$^{+/+}$ and luCldn-18$^{-/-}$mice by qRT-PCR. Gene expressions were normalized to GAPDH. Results are expressed as mean ± SD of 15 to 25 mice. *, p < 0.05. (C) Immunofluorescence micrographs for Cldn18 (green) co-stained with E-cadherin (red) and DAPI (blue) in the lungs from luCldn-18$^{+/+}$ and luCldn-18$^{-/-}$ mice. Representative images of five mice are shown. Scale bars = 20 μm.
